# The Role of Adjuvant Primary Site Radiotherapy for Cutaneous Melanoma Patients with Microsatellitosis

**DOI:** 10.1245/s10434-026-19819-3

**Published:** 2026-05-26

**Authors:** Anjali Devireddy, Yifan Yi, Kelsey N. Nordlund, Ruitao Lin, Aya Salem, Noel X. Yang, Sydney Keatts, Oriana Jerez, Ahsan Farooqi, Andrew J. Bishop, B. Ashleigh Guadagnolo, Priyadharsini Nagarajan, Roi Weiser, Sarah B. Fisher, Ryan Goepfert, Merrick I. Ross, Rodabe N. Amaria, Isabella C. Glitza Oliva, Alison K. Yoder, Devarati Mitra

**Affiliations:** 1 Department of Radiation Oncology, The University of Texas MD Anderson Cancer Center, Houston, TX USA; 2 Department of Biostatistics, The University of Texas MD Anderson Cancer Center, Houston, TX USA; 3 Department of Anatomical Pathology, The University of Texas MD Anderson Cancer Center, Houston, TX USA; 4 Department of Surgical Oncology, The University of Texas MD Anderson Cancer Center, Houston, TX USA; 5 Department of Head and Neck Surgery, The University of Texas MD Anderson Cancer Center, Houston, TX USA; 6 Department of Melanoma Medical Oncology, The University of Texas MD Anderson Cancer Center, Houston, TX USA

## Abstract

**Purpose:**

The presence of microsatellites in cutaneous melanoma has historically been associated with a poor prognosis. Whether adjuvant primary site radiation therapy (RT) mitigates risk of recurrence is unknown.

**Methods:**

All patients with cutaneous melanoma who underwent primary site wide local excision at our center between 2016 and 2023 with histopathologic evidence of microsatellites and no clinically involved lymph nodes, in-transit disease, or macrosatellites were retrospectively reviewed. We investigated the association of patient, disease, and treatment factors with recurrence and survival outcomes.

**Results:**

We identified 153 eligible patients with microsatellites. With a median follow-up of 31 months from resection, 3-year local recurrence-free survival (LRFS) was 78% and 3-year disease-free survival (DFS) was 42%. The majority received adjuvant systemic therapy (55%, n = 85 immune checkpoint inhibition; 5%, n = 7 BRAF/MEK-directed therapy). Fifty-nine patients (39%) received postoperative RT after wide local excision. On multivariate analyses with inverse propensity score weighting, receipt of adjuvant primary site RT was associated with longer local recurrence-free survival (LRFS, hazard ratio 0.22, *p* = 0.04), which appears to drive longer disease-free survival (DFS, hazard ratio 0.46, *p* = 0.007).

**Conclusions:**

In the contemporary therapeutic era, even with the majority of patients receiving adjuvant immune checkpoint inhibition or targeted therapy, adjuvant RT is associated with higher local control and DFS. Patients with microsatellitosis should be referred for consideration of postoperative RT.

**Supplementary Information:**

The online version contains supplementary material available at 10.1245/s10434-026-19819-3.

Microsatellites represent discontinuous nests of melanoma cells, typically identified in the primary tumor resection specimen, and thus existing within a few centimeters of the primary tumor.^1^ Prior studies dating back to the 1980s and 1990s have shown the presence of microsatellites is associated with higher likelihood of diagnosis with regional disease and worse prognosis overall.^2–4^ In this context, microsatellites (even in the absence of of other regional disease) have been an indication of Stage III melanoma since the 6^th^ AJCC edition.^5^

Microsatellites are also known to increase the risk of locoregional recurrence, although the effect on local recurrence specifically is less well characterized. A prior study of 98 patients with microsatellites undergoing sentinel lymph node biopsy found isolated locoregional recurrence was the most common site of first recurrence (27% of 41 recurrences) with non-in-tranist (presumably mostly local) subcutaneous disease representing 20% of recurrence events overall.^6^ Interestingly, several studies have specifically found that microsatellites are significantly associated with locoregional recurrence but not distant metastasis, including a study of 68 patients with microsatellites of whom 54% experienced locoregional recurrence and a study of 45 patients with microsatellites of whom 55% experienced a regional nodal recurrence and 35% experienced a macrosatellite (presumably local or in transit) recurrence.^7,8^ One study specifically evaluated local recurrence in a cohort of 60 patients with ≥3-mm thick primary tumors and found that local recurrence was greater in those with vs. without microsatellites (14% vs. 3%).^9^

Adjuvant systemic therapies such as immune checkpoint inhibition and BRAF-targeted therapies have changed management options for patients with Stage III disease in the contemporary era. However, the specific effect of these treatments on patients with microsatellites is not well characterized. KEYNOTE 54 and CheckMate 238 (evaluating adjuvant anti-PD1) and COMBI-AD (evaluating adjuvant dabrafenib/trametinib) showed overall relapse-free survival benefits to the experimental treatment arm, though did not report on prevalence of microsatellites. KEYNOTE 54 specifically required regional nodal disease as a criterion for enrollment.^10–12^ KEYNOTE 716 evaluated adjuvant pembrolizumab for Stage IIB or IIC disease though 5 of the 1,182 patients enrolled carried N1c disease, which implies in transit, satellite, or microsatellite disease.^13^ Thus, the effect of contemporary systemic therapy on recurrence risk specifically for microsatellite disease (in the absence of nodal disease) is unknown, although would be expected to be similarly advantageous as in other locoregionally advanced clinical contexts.

The role of adjuvant primary site radiotherapy is unclear in the management of patients with microsatellites. For melanoma broadly, prior data suggests that for all-comers with T1b-T4b disease, local recurrence (LR) can be a significant issue when pathologic specimens contain high risk features, with >10% experiencing LR (defined as recurrence within 2 cm of the surgical site).^14–17^ Studies have shown that LR is associated with poor overall outcomes including higher likelihood of regional and distant metastases as well as decreased overall survival.^18^ Additionally, LR requires further therapy including re-resection with or without systemic therapy and/or RT. Each additional modality can increase the potential for treatment-associated morbidity, including cosmetic or functional consequences to re-resection and at least some degree of toxicity expected in most patients receiving anti-PD1 therapy.^14^

Historically, RT after WLE has been utilized to decrease LR risk in patients at elevated risk.^19–21^ However, the data supporting adjuvant RT predates the contemporary therapeutic era. Thus, it is unknown whether there continues to be a benefit of RT after WLE, sentinel lymph node biopsy, and modern adjuvant systemic therapy. In this study, we evaluated the outcomes of a large cohort of patients with melanoma microsatellitosis managed in the modern treatment era, with specific focus on the association of RT with patterns of recurrence and disease control.

## Methods

### Patient Cohort

We queried our institutional database and identified 189 cutaneous melanoma patients with microscopic satellite metastases (microsatellites) who underwent WLE at our institution between 2016 and 2023. All pathologic specimens were reviewed by an expert dermatopathologist at our tertiary care referral center. Thirteen patients were excluded for having either macrosatellites or in-transit lesions, 19 were excluded for clinically positive nodal disease on preoperative imaging, and one was excluded for metastasis beyond the lymph nodes. Three patients were excluded for having received neoadjuvant systemic therapy. Thus, 153 patients remained in the final cohort. Review of patient medical records commenced after approval from the institutional review board.

### Defining Disease and Treatment Characteristics

Primary tumor histopathologic features, as described by our institutional dermatopathologists, were collected for all patients in the cohort. Mutational testing was performed using targeted markers (n = 23) or a next generation sequencing panel (n = 115) and was performed at the time of surgical resection, prior to initiation of adjuvant systemic therapy. Adjuvant systemic therapy was defined as therapy administered after the last oncologic resection during the initial treatment episode.

### Clinical Follow-up and Assessment of Outcome

Time-to-event calculations were performed from the date of primary surgical resection. Our institutional practice is to recommend follow-up every 3–4 months for 2 years followed by every 6 months through Year 5. Follow-up for patients with microsatellitosis at our institution include physical exam and cross-sectional imaging of the chest, abdomen, pelvis ± neck (if the primary tumor was diagnosed in the head and neck area). Brain MRI is typically performed at baseline and at the time of detected recurrence. Local recurrence (LR) was defined as recurrence within 2 cm of the surgical incision. In-transit recurrence (ITR) was defined as recurrence beyond LR but proximal to first echelon draining nodal basins. Nodal recurrence (NR) was defined as recurrence within a primary draining nodal basin. Distant metastasis (DM) was defined as recurrence beyond local and regional/in-transit recurrence. Disease-free survival (DFS) was defined as the time from resection to last follow-up without evidence of disease.

### Statistical Methods

Categorical variables were compared between the cohorts receiving adjuvant primary site radiotherapy (RT) and no adjuvant primary site radiotherapy (no RT) using the chi-squared test, and continuous variables were compared using the Mann–Whitney *U* test. The Kaplan–Meier method with Greenwood's formula was used to estimate local recurrence-free survival (LRFS), distant metastasis-free survival (DMFS), and DFS. Unadjusted and inverse probability of treatment weighting (IPW)-adjusted log-rank tests were performed to assess survival differences between the RT and no RT groups. The propensity score was estimated using a logistic regression model with baseline patient and disease factors weighted to balance covariates between groups. Covariate balance was assessed using standardized mean differences (within 0.1) and Love plots. IPW-adjusted Cox regression models were further fitted to derive covariate-adjusted effects of radiotherapy on LRFS and DFS. All analyses were conducted using R version 4.5.1.

## Results

### Patient Cohort and Primary Disease

Patient and primary tumor characteristics, divided by receipt of adjuvant RT to the primary site, are shown in Table 1. Ninety-four patients (61%) did not receive RT, whereas 59 (39%) received RT. There were no statistically significant differences in sex, primary tumor site, histologic subtype, ulceration, perineural invasion, or median Breslow thickness for patients who received or did not receive RT. The most common anatomic site was the head and neck. Adverse pathologic features were common and included ulceration (39% of those not receiving RT and 51% of those receiving RT) and perineural invasion (36% of those not receiving RT and 44% of those receiving RT). Lymphovascular invasion was more common in patients not receiving RT (66% vs. 46%, *p* = 0.01). Mutational testing was performed for most patients with just over half of those evaluated carrying an oncogenic BRAF mutation in both cohorts.

**Table 1:** Patient and primary disease characteristics

Median age at primary tumor excision (IQR)	No adjuvant primary site RT (n = 94)		Adjuvant primary site RT (n = 59)		*p*-value
	65 (51–75)		69 (60.5–76.5)		0.08
	n	% no RT	n	% RT	
Sex					0.35
Female	34	36%	17	29%	
Male	60	64%	42	71%	
Primary site				0.51	
Head and neck	29	31%	25	43%	
Trunk	28	30%	13	22%	
Upper extremity	16	17%	9	15%	
Lower extremity	22	22%	12	20%	
Histologic melanoma subtype			0.75		
Superficial spreading	41	44%	25	42%	
Nodular	34	36%	22	37%	
Lentigo maligna	6	6%	5	9%	
Acral lentiginous	7	8%	2	3%	
Not specified	6	6%	5	9%	
Ulceration	37	39%	30	51%	0.16
Perineural invasion	34	36%	26	44%	0.33
Lymphovascular Invasion	62	66%	27	46%	0.01
Median Breslow thickness (IQR)	3.7 mm (1.8-6.8 mm)		4.6 mm (2.7-7.2 mm)		0.09
Tumor mutations	n	% tested (# tested)	n	% tested (# tested)	
BRAF	43	52% (82)	29	52% (56)	0.94
NRAS	18	27% (66)	8	15% (53)	0.11
KIT	5	8% (65)	3	6% (53)	1.00

*IQR* interquartile range; *RT* radiation therapy; *n* number of patients

### Treatment Characteristics: Surgery

Primary site and nodal surgery details are shown in Table 2. Seven patients (4%) had positive surgical resection margins; three received adjuvant RT. Nodal evaluation was performed for 89% of patients without adjuvant primary site RT and 93% receiving RT. Three patients underwent elective nodal dissection but the remainder of nodal evaluations consisted of sentinel lymph node biopsy. An involved sentinel node was found in 66% of patients in the overall cohort for whom surgical nodal evaluation was performed (n = 137) with no significant difference between those who did not vs. did receive RT. There was a median single node involved with or without adjuvant primary site RT and the median nodal deposit size was 3.0 mm for patients not receiving adjuvant primary site RT and 2.0 mm for those receiving RT.

**Table 2:** Treatment characteristics

	No adjuvant primary site RT (n = 94)		Adjuvant primary site RT (n = 59)		*p*-value
	n	% no RT	n	% RT	
*Surgical therapy*					
Surgical margin status					1.00
Negative	90	96%	56	95%	
Positive	4	4%	3	5%	
Sentinel lymph node biopsy	84	89%	55	93%	0.42
	n	% LN eval (n = 83)	n	% LN eval (n = 54)	
LN+	58	70%	33	61%	0.27
ECE	15	18%	6	11%	0.25
Median LN removed (IQR)	3 (2–14)		2 (2–4)		0.26
Median LN involved (IQR)	1 (0–2)		1 (0–2)		0.23
Median LN deposit size (IQR)	3 mm (1.2–6.3 mm)		2 mm (1.1–3 mm)		0.37
*Systemic therapy*					
Adjuvant systemic therapy					0.23
Immunotherapy	48	51%	37	63%	
Targeted therapy	6	6%	1	2%	

*eval* evaluated; *IQR* interquartile range; *LN* lymph node; *n* number of patients; *RT* radiation therapy

### Treatment Characteristics: Systemic Therapy

Ninety-two patients (59%) received adjuvant systemic therapy (Table 2). Most patients received immune checkpoint inhibition (n = 85, 55%). Single-agent anti-PD1 was the most common regimen; 65 patients (42%) received nivolumab, and 12 (8%) received pembrolizumab. Four patients (3%) received combination therapy with ipilimumab and nivolumab. Seven patients (5%) received adjuvant dabrafenib and trametinib. Of note, most oncogenic BRAF-carrying patients received immune checkpoint inhibition (n = 47, 65% of BRAF-positive patients), whereas only six patients (8%) received targeted therapy.

#### Treatment Characteristics: Radiation Therapy

Fifty-nine patients received adjuvant primary site RT (39%). The median time from surgery to RT was 8 weeks (interquartile range [IQR] 6–10 weeks). In all cases, 5 × 6 Gy was prescribed to Dmax with a goal of 90% prescription dose (27 Gy) covering the target field. Radiotherapy fractions were delivered either every other business day or twice a week. Modalities included electrons (n = 48), 3D photons (n = 3), or IMRT/VMAT (n = 8). Of note, adjuvant RT was more common in recent years (16%, 2016–2018 vs. 55%, 2019–2023, *p <* 0.001).

Given the absence of clinically involved nodal disease, nodal RT was uncommon (n = 12, 7.8%) but was exclusively delivered to patients who were also receiving adjuvant primary site RT (20% of primary RT patients vs. 0% no primary RT, *p <* 0.001). Of those receiving nodal RT, there was a median of two sentinel nodes involved (IQR 1–3) with a median largest deposit size of 3.35 mm (IQR 1.75–11.63). Four of these patients had extracapsular extension.

### Clinical Outcomes

Median follow-up for the overall cohort was 31 months (IQR 19.5–50.5). There was no significant difference in follow-up between patients who lived <100 miles from our center (30.4 months) and those who lived >100 miles from our center (31.3 months; *p* = 0.77). Because adjuvant RT was more common in recent years, there was a trend to longer follow-up time for patients who did not receive RT (median 34.9 months vs. 27.3 months, *p* = 0.054). Sixty-four percent of patients in the overall cohort (n = 83) had 2-year follow-up data available. This was similar between those receiving RT (61%; 36 of 59 total) and those not receiving RT (66%; 62 of 94 total). Follow-up time also did not significantly differ for patients who lived further from our center within the subgroups of those who received RT (27.1 months <100 miles vs. 27.3 months >100 miles; *p* = 0.66) vs. those who did not receive RT (36.5 months <100 miles vs. 31.9 months >100 miles; *p* = 0.52). Thus, there was no significant interaction between RT and distance to our center overall (*p* = 0.35).

Three-year actuarial outcomes of the overall cohort were 78% (IQR 71–86%) local recurrence-free survival (LRFS), 55% (IQR 34–51%) distant metastasis-free survival (DMFS), and 42% (IQR 27–66%) DFS. Given the cohort who received RT had a median follow-up closer to 2 years, we also evaluated 2-year actural outcomes of the overall cohort and found 84% LRFS (IQR 78–90%), 71% DMFS (IQR 63–79%), and 50% DFS (IQR 42–59%). The median disease-free time was 17.8 months (95% confidence interval [CI] 8.5–25.9) for patients who did not receive RT and 40.7 months (95% CI 22.0-NA) for patients who received RT. Because of the unequal follow-up between cohorts, we also evaluated the overall patient population at the 2-year index timepoint. Specifically we found that 64% of patients overall had 2-year follow-up data (83 of 153 patients), which included 61% of patients receiving adjuvant primary site RT (36 of 59 patients) and 66% of patients not receiveing adjuvant primary site RT (62 of 94 patients).

Patterns of disease recurrence are summarized in Table 3. Having an isolated local recurrence as the first recurrence was seen in 17% of patients (n = 16) who did not receive adjuvant RT vs. 5% of patients (n = 3) treated with RT. Nodal and/or in transit metastases as the first site of recurrence in the absence of distant metastases were also common (32% without RT vs. 20% with RT). Only two patients with nodal and/or in-transit recurrence as the first site of progression had a synchronous local recurrence event. Distant metastasis as a solitary site of first recurrence was relatively uncommon with 16% experiencing this event without RT and 14% with RT.

**Table 3:** Patterns of recurrence

	No adjuvant primary site RT (n = 94)		Adjuvant primary site RT (n = 59)	
	n	% no RT	n	% RT
*First recurrence*				
Local only	16	17%	3	5%
Regional (excluding DM)	30	32%	12	20%
Distant metastasis	15	16%	8	14%
*Location of recurrence at anytime*				
Including local	24	26%	5	9%
Including nodal	40	43%	15	25%
Including in-transit	50	53%	19	32%
Including distant	61	65%	23	39%

*eval* evaluated; *IQR* interquartile range; *LN* lymph node; *n* number of patients; *RT* radiation therapy

The median time to local recurrence was 9.7 months (IQR 3.2–20.2) among patients who experienced a local recurrence. The time to local recurrence was 15.5 months for those receiving adjuvant primary site RT and 8.3 months without RT (*p* = 0.25). Similarly, the median time to any recurrence for those with recurrence was 7.4 months (IQR 3.5–17.7). The time to first recurrence was 9.8 months for those receiving adjuvant primary site RT and 6.2 months without RT (*p* = 0.18).

Among patients who developed a local recurrence, five had received adjuvant radiation therapy and 24 had not. The median time from local recurrence to distant metastasis or last follow-up was 20.4 months (95% CI 20.37–NA) in the RT group and 17.9 months (95% CI 6.18–NA) in the non-RT group (log-rank *p*-value = 0.7).

### Predictors of Outcomes

Table 4 summarizes univariate predictors of LRFS and DFS without adjustment for covariates. The use of adjuvant primary site RT was associated with higher LRFS and DFS (2-year LRFS 92% vs. 78% and 3-year LRFS 89% vs. 72%, *p* = 0.013; 2-year DFS 63% vs. 42% and 3-year DFS 60% vs. 32%, *p* = 0.0027) (Supplemental Fig. 1). On unadjusted analysis, patients who received adjuvant systemic therapy had numerically higher DFS (3-year DFS: targeted therapy 69% vs. immunotherapy 47% vs. none 29%), but this was not a statistically significant difference.

**Table 4:** Unadjusted univariate patient and treatment variable association with outcome

	3-year LRFS	LRFS *p*-value	3-year DMFS	DMFS *p*-value	3-year DFS	DFS *p*-value
*Sex*						
Male	78%	0.67	52%	0.14	40%	0.71
Female	76%		62%		44%	
*Age, years*						
<65	79%	0.86	53%	0.63	46%	0.53
≥65	76%		58%		37%	
*Anatomic site of primary*						
H&N	70%	0.21	59%	0.92	41%	0.57
Trunk	83%		55%		46%	
Upper	72%		50%		48%	
Lower	86%		54%		34%	
*Breslow thickness (mm)*						
≤4	77%	0.29	61%	0.34	44%	0.6
>4	80%		51%		39%	
*Ulceration*						
Absent	77%	0.66	58%	0.42	44%	0.38
Present	79%		51%		38%	
*PNI*						
Absent	82%	0.21	60%	0.24	44%	0.42
Present	72%		48%		37%	
*LVI*						
Absent	74%	0.63	63%	0.1	48%	**0.037**
Present	81%		50%		37%	
*SLN+ at diagnosis*						
No	80%	0.51	61%	0.22	47%	0.073
Yes	81%		51%		42%	
*Oncogenic BRAF*						
Absent	78%	0.80	49%	0.98	34%	0.64
Present	78%		54%		43%	
*Adjuvant systemic therapy*						
None	77%	0.85	49%	0.33	29%	0.15
Immunotherapy	78%		58%		47%	
Targeted therapy	83%		83%		69%	
*Treatment era*						
2016–2018	82%	0.27	52%	0.1	36%	0.17
2019–2023	75%		58%		46%	
*Adjuvent primary site RT*						
No	72%	**0.013**	52%	0.1	32%	**0.0027**
Yes	89%		58%		60%	

*DFS* disease-free survival; *DMFS* distant metastasis-free survival; *LN* lymph node; *LRFS* local recurrence-free survival; *LVI* lymphovascular invasion; *PNI* perineural invasion; *SLN* sentinel lymph node (*p <* 0.05)

**Fig. 1:**
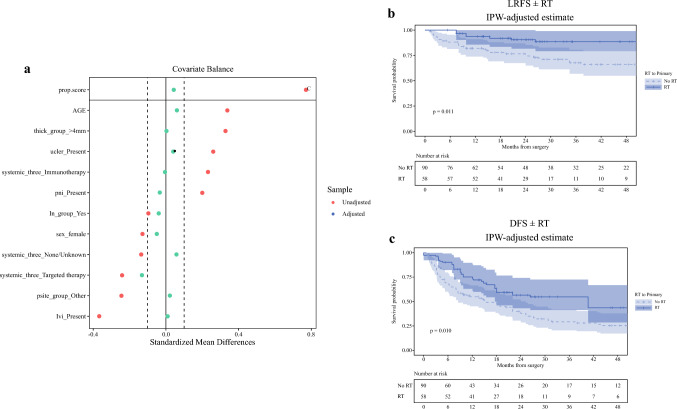
Inverse propensity score weighting confirms that adjuvant primary site radiotherapy improves local recurrence-free survival and disease-free survival. **a** Covariate balance was assessed by using standard mean differences (within 0.1) and Love plots. Before adjustement several variables, such as treatment era, age, and tumor thickness showed marked imbalance, but after weighting, all covariates had standardized mean differences below 0.1. **b** Adjuvant primary site RT improves LRFS (*p* = 0.011). **c** Adjuvant primary site RT improves DFS (*p* = 0.01)

We then undertook inverse propensity-weighting (IPW)-adjusted analyses to evaluate if the association of RT with higher LRFS and DFS held up upon balancing for patient and disease factors (Fig. 1a). On univariate analysis we were able to confirm that this was the case (Figs. 1b–c). Subsequently, we generated a IPW-adjusted multivariate Cox proportional hazards model to evaluate possible predictors of LRFS and DFS (Table 5). Upon balancing covariates, adjuvant primary site RT continued to be associated with higher LRFS (hazard ratio [HR] 0.22, 95% CI 0.05–0.93, *p* = 0.04) and higher DFS (HR 0.46, 95% CI 0.26–0.81, *p* = 0.007) (Table 5).

**Table 5:** IPW-adjusted multivariate Cox models for local recurrence and disease free survival

Local recurrence-free survival			Disease-free survival		
	HR (95% CI)	*p*-value		HR (95% CI)	*p*-value
Sex			Sex		
Female	1.24 (0.55–2.78)	0.6	Female	0.93 (0.57–1.54)	0.79
Male	Reference 1.01 (0.98–1.04)	0.61	Male	Reference 1.01 (0.99–1.03)	0.28
Age			Age		
Primary site			Primary site		
Head and neck	2.41 (0.94–6.2)		Head and neck	1.25 (0.69–2.27)	0.46
Other	Reference		Other	Reference	
Thickness (mm)			Thickness (mm)		
>4	0.66 (0.31–1.42)	0.29	>4	1.06 (0.62–1.79)	0.984
$\le$ 4	Reference		≤4	Reference	
Ulceration			Ulceration		
Present	1.86 (0.68–5.1)	0.23	Present	1.51 (0.93–2.46)	0.091
Absent	Reference		Absent	Reference	
PNI			PNI		
Present	2.59 (1.03–6.49)	0.043	Present	1.26 (0.73–2.17)	0.41
Absent	Reference		Absent	Reference	
LVI			LVI		
Present	1.99 (0.86–4.63)	0.11	Present	0.79 (0.5–1.26)	0.33
Absent	Reference		Absent	Reference	
SLN+			SLN+		
Yes	1.09 (0.53–2.25)	0.82	Yes	0.72 (0.45–1.17)	0.18
No	Reference		No	Reference	
Systemic therapy			Systemic therapy		
Immunotherapy	1.48 (0.65–3.37)	0.35	Present	0.70 (0.42–1.17)	1.17
Targeted therapy	1.09 (0.29–6.3)	0.92	Targeted therapy	0.46 (0.14–1.51)	0.20
None	Reference		None	Reference	
RT to primary site			RT to primary site		
Yes	0.22 (0.05–0.93)	0.040	Yes	0.46 (0.26–0.81)	0.007
No	Reference		No	Reference	

*CI* confidence interval; *HR* hazard ratio; *LVI* lymphovascular invasion; *PNI* perineural invasion; RT radiation therapy, *SLN+* sentinel lymph node positive (*p <* 0.05)

## Discussion

This study represents the largest cohort, to our knowledge, investigating the use of adjuvant primary site RT for cutaneous melanoma patients with microsatellites treated in the contemporary therapeutic era. Overall, we found that patients with microsatellitosis continue to have high rates of disease recurrence, even with 59% receiving immune checkpoint inhibition or BRAF/MEK targeted therapy. The most likely site of first recurrence for patients with microsatellite disease was local, with 25% of patients (n = 19) in this cohort presenting with local-only disease as their first site of recurrence.

Given this elevated risk of local recurrence despite wide local excision and the availability of effective adjuvant systemic therapy, we sought to evaluate the association of adjuvant RT with outcomes. We found that local recurrence-free survival was significantly higher for patients who received adjuvant primary site RT (3-year LRFS 89% vs. 72%, *p* = 0.015), and this effect held up on IPW-adjusted multivariate modeling (HR 0.17, *p* = 0.028). However, given that radiotherapy for locoregionally confined melanoma has never previously been associated with improved overall outcomes, we were surprised to find that patients who received RT also experienced higher DFS (3-year DFS 59% vs. 34%, *p* = 0.007), which similarly held up on IPW-adjusted multivariate modeling (HR 0.5, *p* = 0.022). Of note, in the absence of RT 20% of patients developed local only recurrence as their first site of disease progression, whereas with RT only 5% of patients develop local only disease as their first site of recurrence. One may hypothesize that adjuvant RT delays the time to any recurrence by significantly decreasing the likelihood of local recurrence at the primary surgical site, which in the context of microsatellites is high. Further support for locoregional recurrence driving DFS comes from the observation that the likelihood of distant metastasis as a first site of recurrence was similar with or without RT (16 vs. 15%). This is consistent with others who have found that the presence of microsatellites specifically increases the likelihood of locoregional recurrence and shortens DFS without as significant an effect on distant metastasis.^7,8^

Establishing the role of RT for melanoma patients with microsatellites also depends on evaluating the toxicities of treatment in the context of the downstream consequences of forgoing treatment. Most patients receiving adjuvant RT will develop grade 1–2 acute dermatitis in the radiotherapy field that resolves a few weeks after treatment completion. The RTN2 Trial evaluating adjuvant RT vs. observation for neurotropic melanoma found no patients experienced grade 3+ acute toxicity with grade 3 late toxicity experienced by 10% of patients in the observation arm and 12.5% of patients in the RT arm.^22^ Overall, this suggests that RT is very well tolerated with low likelihood of long-term toxicity. For patients who develop local recurrence, the typical treatment pathway involves re-resection, which can be cosmetically or functionally compromising and can necessitate loss of a previously placed graft and/or flap reconstruction. There may also be the consideration of systemic therapy such as immune checkpoint inhibition. CheckMate 238 evaluating adjuvant nivolumab vs. ipilimumab found 96.9% of patients receiving nivolumab experienced any adverse event with 14.4% developing a grade 3+ event.^11^ This is similar to the 93.3% of patients receiving pembrolizumab in the KEYNOTE-054 study experiencing any degree of toxicity with 14.7% having a grade 3+ event.^10^ Should a patient subsequently require combination anti-PD1/anti-CTLA4 therapy, the expected grade 3+ toxicity rate may be greater than threefold higher than anti-PD1 alone.^23^ Thus, by delaying time to melanoma progression, adjuvant RT has the potential to delay the need to implement more aggressive surgical or systemic therapy.

Prior to this study, there was a lack of data evaluating the association of adjuvant RT with disease control for melanoma patients with microsatellites. However, there is a precedent for RT broadly being helpful in decreasing local recurrence rates for high-risk patients.^20,24^ The most robust prior data exists in support of RT for patients with positive margin resection or desmoplastic melanoma but we hope the data presented here serves to further suggest that adjuvant primary site RT has an important role in the management of high-risk locoregionally confined cutaneous melanoma.

Of note, while most patients in this cohort received adjuvant systemic therapy, not all patients did so. In contemporary practice at our institution, adjuvant systemic therapy is typically discussed for eligible patients with IIB-III disease. However, particularly for patients without nodal disease (30% of our cohort) or microscopic nodal disease measuring <1 mm in a single node, there are detailed discussions of risk/benefit for adjuvant systemic therapy, especially as there is no known overall survival benefit to adjuvant anti-PD1 or BRAF/MEK-directed therapies. Thus, with successful salvage possible at recurrence, and the possibility of life-altering side effects from treatment, a significant number of patients opt to forgo adjuvant systemic therapy and instead continue under close surveillance. Thus, the heterogeneity of adjuvant systemic therapy administration of this study reflects a real-world heterogeneity of administration for patients with microsatellites without clinically involved nodes, in transits, or macrosatellites.

A primary limitation of our conclusions is the retrospective nature of our study. Selection bias likely influenced who received postoperative RT and adjuvant systemic therapy. However, given that higher risk patients would typically be selected for treatment intensification, this selection bias would not be expected to negate our findings. Another limitation that arises in the context of the retrospective nature of our study is the fact that it is impossible to ensure every patient undergoes every 3- to 4-month recommended follow-up appointment. However, in general, with a median follow-up of a little more than 3 years, we have found that most patients come to our center as recommended for surveillance imaging and physical exam as recommended until their last follow-up visit (at which time they often transfer care locally). There are also likely unaccounted for risk factors not incorporated in our model that may explain why systemic therapy was not statistically associated with longer DFS. Of note, however, there was a mild trend to prolonged DFS with HR of 0.46 and 0.7 for targeted therapy and immunotherapy, respectively (*p* = 0.17–0.2). In addition, while there was unequal follow-up for patients who did or did not receive RT, given that the majority of recurrence events occurred within the first 2 years, this difference is unlikely to change the overall conclusion. We hypothesize that this difference emerged as a product of evolving referral patterns at our institution in the context of tighter integration of the radiation oncology group within the melanoma center and the observation that anecdotally patients with microsatellites were observed having high likelihood of local recurrence such that there was increasing interest in more aggressive local control strategies. Of note, in this study, involvement of a sentinel node was not associated with overall outcomes, which was unexpected given the well established data that nodal disease is associated with worse prognosis. Data from the Sentinel Lymph Node Working Group (SLNWG) recently found that in a cohort of >13,000 patients undergoing SLNB, of whom 426 had nonnodal regional disease at diagnosis (including microsatellites), SLN involvement was associated with worse RFS, even with nonnodal regional disease.^25^ One confounding variable in our dataset was the use of adjuvant systemic therapy, which was received by 69% of patients with an involved node vs. 48% of patients in the absence of an involved node (*p* = 0.02). Thus, adjuvant systemic therapy likely offset some of the increased risk of recurrence events in the context of nodal involvement. This difference as well as our relatively limited cohort size may have masked an overall effect of nodal disease on DFS. Supporting this hypothesis was the observation that on univariate modeling in our cohort there was a trend to association of SLN status with DFS (3-year DFS 47% vs. 42%, *p* = 0.073).

Overall, our data suggest that even in the contemporary therapeutic era, adjuvant primary site RT decreases the risk of local recurrence. Additionally, our data suggest that RT may delay time to overall disease progression. Given the favorable toxicity profile of postoperative RT, these data support the use of postoperative primary site RT for patients with cutaneous melanoma found to have microsatellites and no evidence of distant disease beyond microscopic nodal metastasis. Patients with these pathologic features should be referred for consideration of postoperative RT.

## Supplementary Information

Below is the link to the electronic supplementary material.

Supplementary file1 (PPTX 12310 kb)
