# Supplementary figures and images for: The Role of Adjuvant Primary Site Radiotherapy for Cutaneous Melanoma Patients with Microsatellitosis
Source: Ann Surg Oncol. 2026 May 26;33(8):7461–70. doi: 10.1245/s10434-026-19819-3 (PMC13337811; doi:10.1245/s10434-026-19819-3)

## Slide 1
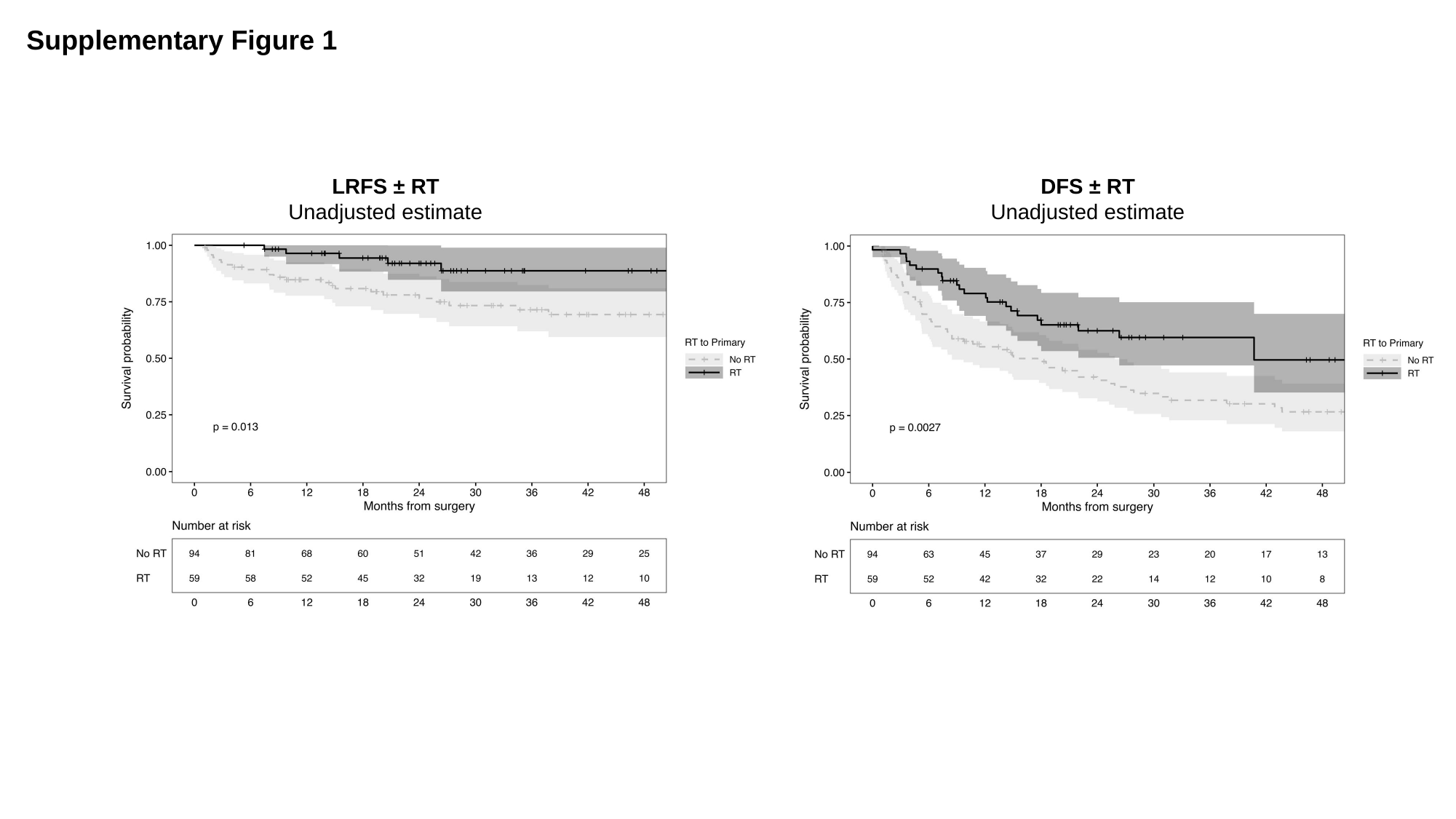

Supplementary Figure 1
LRFS ± RT
Unadjusted estimate
DFS ± RT
Unadjusted estimate

Supplement: Supplementary file 1 — Supplementary file1 (PPTX 12310 kb) [file 10434_2026_19819_MOESM1_ESM.pptx]
